# GA-BP-Based Low-Noise FBG Hydroacoustic Monitoring System with Reference Sensor

**DOI:** 10.3390/s24175733

**Published:** 2024-09-04

**Authors:** Yubin Zhou, Yuexia Zhao, Chengbing Song, Jiancun Wang, Weikun Xu, Zhengguang Li

**Affiliations:** 1 National Deep Sea Center of China, Qingdao 266237, China; 2 Laboratory for Marine Geology, Qingdao National Laboratory for Marine Science and Technology, Qingdao 266237, China

**Keywords:** fiber Bragg grating, hydroacoustic monitoring, GA-BP, fiber-optic hydrophone

## Abstract

To address the issue of harsh marine background noise impacting the monitoring signal of fiber-optic hydrophones, we propose a low-noise fiber Bragg grating (FBG) hydroacoustic monitoring system with a reference sensor based on genetic algorithm backpropagation (GA-BP). Through theoretical analysis, we deduce the noise suppression steps of the GA-BP algorithm based on the reference sensor and construct train and test sets based on the data from the reference sensor and monitoring sensor at different times, optimizing the GA-BP algorithm to find the best fitting results and thereby obtaining the low-noise monitoring signal. Experimental results from the anechoic tank show that the proposed method can suppress background noise interference on effective signals and that the suppression effect improves as the background noise increases. The sound pressure sensitivity ranges from −173.76 dB to −171.33 dB at frequencies of 8 kHz to 12 kHz, with a response flatness of less than 2.43 dB. The noise suppression effect is obvious under the condition of poor signal-to-noise ratio (SNR), which can reach more than 18.3 dB. The advantages of the proposed algorithm in array signal processing are further demonstrated by the directivity experiment, which proves that the algorithm has a great potential for engineering applications in harsh marine environment.

## 1. Introduction

Marine environmental monitoring is of great significance in scientific investigation [1]. Common ocean monitoring sensors are divided into various types, including temperature [2], salinity [3], depth [4], pH [5], and vibration [6]. Among them, hydroacoustic signal monitoring technology is particularly useful for detecting seabed geological structures, assessing seismic activity, and exploring seabed resources. This technology has been extensively studied worldwide in recent years [7]. Traditional hydrophones typically use piezoelectric materials to respond to hydroacoustic signals to realize hydroacoustic signal monitoring, offering advantages such as simple structure and high reliability. However, due to the limitations of their sensing principle, piezoelectric sensors struggle with multiplexing monitoring [8]. In contrast, fiber-optic hydrophones use phase changes or light intensity changes in the fiber to sense sound waves, and have the characteristics of anti-electromagnetic interference [9]. Additionally, due to low transmission loss, they can achieve long-distance monitoring and large-scale reuse [10].

High signal-to-noise ratio (SNR) with reflected light can be obtained by writing fiber Bragg grating (FBG) on the signal mode fiber (SMF), thereby obtaining high-quality sensing signals [11]. When the reflectivity is sufficiently low, a sensor monitoring array with thousands of primitive elements can be prepared by time division multiplexing (TDM) technology [12,13]. Recent experiments have also proved the effectiveness of FBG hydrophones for marine environmental monitoring in recent years. Wu et al. [14] proposed a hydrophone towed line array with a diameter of 1.7 mm based on ultra-weak fiber Bragg grating (uwFBG). However, the marine environment contains a significant amount of background noise [15], such as the sounds of ship engines, wave impacts, and the marine organism activity [16,17]. These noises can confuse or cover up the characteristics of monitoring signals, increasing the difficulty of signal detection by fiber-optic sensors [18].

To improve the acoustic signal monitoring effectiveness of fiber-optic hydrophones and reduce the impact of noise on the system, researchers have conducted in-depth exploration, which can be mainly divided into two aspects. On the one hand, they optimize the sensing principle through the optical path. For instance, Ma et al. [19] isolate high-frequency noise through the design of the sensing probe to resist high-frequency environmental noise interference. However, the structural design typically achieves noise reduction only for a specific frequency band. For achieving full-band noise reduction, Cai et al. [20] demodulated sensing signals and noise signals by setting two 3 × 3 interferometers, respectively, and proved that the proposed demodulation method can reduce harmonic distortion signals and improve SNR effectively. The method has obvious advantages in noise reduction. On the other hand, researchers also use algorithms to improve the signal SNR of fiber-optic hydrophones. Pang et al. [21] corrected the noise introduced by interferometer defects through preprocessing of interference signals, proving that the preprocessing algorithm significantly improves SNR. Moradi et al. [22] combined the phase generation carrier (PGC) algorithm with rotation digital computer to enhance the SNR of the system. It is more conducive to the demodulation of computer software and the actual monitoring of the ocean sound field.

The underwater sound field in the marine environment is relatively complex. At the same time, fiber-optic hydrophones have a wide range of self-noise sources, which together contribute to the random and nonlinear characteristics of the signal [18]. In recent years, machine learning, as an important branch of emerging artificial intelligence technology, has provided a theoretical basis for noise suppression in fiber-optic hydrophones by learning from data and making predictions or decisions [23]. Peng et al. [24] proposed a traffic prediction model based on the genetic algorithm backpropagation (GA-BP) method. This model combines the genetic algorithm and network optimization method, which can improve the training effect and prediction accuracy of neural network in fiber-optic sensing signal, showing the advantages to other competitors. To reduce the measurement error of fiber-optic displacement sensor, Wu et al. [25] designed a compensation and correction model based on GA-BP, proving the effectiveness of the application of machine learning algorithms in improving the measurement accuracy of fiber-optic sensors. Liu et al. [26] used the GA-BP algorithm to improve the spatial resolution of hydrophones. Although this method can be applied to interference-matched fiber-optic hydrophone array, the impact on noise was not further analyzed. The advantage of the GA-BP algorithm lies in its ability to analyze historical data to accurately predict future data, which requires a certain correlation. For fiber-optic sensing temperature measurement technology, current temperature measurement often depends on historical temperature changes [27]. However, in the field of hydroacoustic monitoring, acoustic signal has no clear correlation in time series, which is one of the difficulties in the application of the method in the hydroacoustic field.

The aforementioned research demonstrates that the optimization methods have great potential in fiber-optic sensing signal prediction. However, constructing reasonable training data is key to improving output signal quality. Based on the structural characteristics of the identical FBG hydrophone array studied by our team, to improve the SNR of matching interferometric fiber-optic hydrophone array, in monitoring the ocean sound field, we propose a noise suppression algorithm of the ocean monitoring hydrophone array based on the GA-BP model constructed by the reference sensor. We design an isolated reference sensor for generating the input signal, and the monitoring sensor signal is used, too, as the training set and the test set of machine learning to improve the signal quality of the monitoring signal subsequently.

## 2. Principle

**Figure 1:**
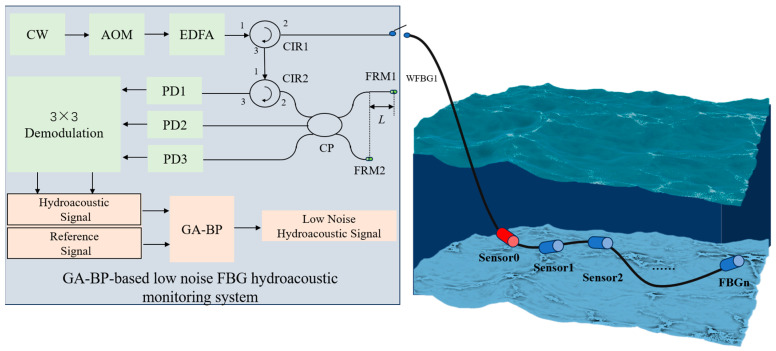
GA-BP-based low-noise FBG hydroacoustic monitoring system.

The schematic diagram for the GA-BP-based low-noise FBG hydroacoustic monitoring system with the reference sensor is shown in Figure 1. The continuous wave (CW) emitted by the narrow linewidth laser is modulated into a pulse wave by an acousto-optic modulator (AOM) and amplified by an erbium-doped optical fiber amplifier (EDFA); it then enters the FBG monitoring array through Circulator 1 (CIR1). The monitoring array is equipped with a reference sensor and hydroacoustic sensors, each composed of a pair of weak-reflection FBGs. The pulsed light is reflected to the CIR1 through the weak-reflection FBG, and then enters the Michelson interferometer through Circulator 2 (CIR2). The Michelson interferometer consists of a 3 × 3 coupler, Faraday rotating mirror 1 (FRM1), Faraday rotating mirror 2 (FRM2), and interferometer arms. It is worth noting that the length of the interferometer arm is inconsistent, with the difference equal to the FBG spacing of the sensors, to achieve matching interference. After the interference signal passes through three photoelectric detectors (PDs), the detection signals from the reference sensor and the hydroacoustic sensor are demodulated by a 3 × 3 demodulation algorithm, selected as the training set and the test set and then input into the GA-BP algorithm model. A low-noise hydroacoustic signal is then obtained.

In theory, the three interference signals of the monitoring sensor_1_ can be expressed as [26]

(1) $$I_{S1}(t)=A+B\cos[\varphi_{s}+\varphi_{n1}+\varphi_{n2}-(z-1)\frac{2\pi }{3}]\;\;(z=1,2,3),$$

where *A* is the direct component of the interference signal, *B* is the AC quantity coefficient of the interference signal, *φ_s_*, *φ_n_*_1_, and *φ_n_*_2_ are the phase generated by hydroacoustic signal, respectively, the phase generated by environmental noise, and the self-noise phase generated by the system light source and other devices.

It has been shown that the special design of the sensing structure can make the fiber-optic hydrophone more sensitive or less sensitive to sound wave responses [28,29]. When the reference sensor is packaged with multi-layer vibration isolation, its ability to receive external environmental noise signals is greatly suppressed. However, the self-noise inside the optical path remains unaffected. In contrast, the monitoring hydroacoustic sensor is affected by self-noise, environmental noise, and target characteristic signal. Therefore, the interference signals of three PDs for reference sensor_0_ can be expressed as

(2) $$I_{S0}(t)=A+B\cos[p\varphi_{s}+q\varphi_{n1}+\varphi_{n2}-(z-1)\frac{2\pi }{3}]\;\;(z=1,2,3).$$

Considering that the vibration isolation level of the reference sensor placed underwater cannot completely eliminate hydroacoustic signals and underwater environmental noise, suppression factors *p* and *q* are taken, respectively, where *p*∈(0, 1) and *q*∈(0, 1). The isolation design effectiveness of the reference sensor is directly related to the value of the suppression factors. The above interference signals are calculated through the 3 × 3 coupler demodulation algorithm to obtain the train set for monitoring sensor phase *φ*_1_ and the reference sensor phase *φ*_0_, respectively, as at the time [*T*_0_, *T*_1_], which can be expressed as

(3) $$\varphi_{1}^{T_{1}}(t)=\sum_{0}^{T_{1}}\varphi_{s}\left(t\right)+\varphi_{n1}\left(t\right)+\varphi_{n2}\left(t\right),$$

(4) $$\varphi_{0}^{T_{1}}(t)=\sum_{0}^{T_{1}}p\varphi_{s}\left(t\right)+q\varphi_{n1}\left(t\right)+\varphi_{n2}\left(t\right).$$

The untreated original monitoring sensor phase *φ*_1_ and the reference sensor phase *φ*_0_, respectively, at the time [*T*_1_, *T*_2_] can be expressed as

(5) $$\varphi_{1}^{T_{2}}(t)=\sum_{T_{1}}^{T_{2}}\varphi_{s}\left(t\right)+\varphi_{n1}\left(t\right)+\varphi_{n2}\left(t\right),$$

(6) $$\varphi_{0}^{T_{2}}(t)=\sum_{T_{1}}^{T_{2}}p\varphi_{s}\left(t\right)+q\varphi_{n1}\left(t\right)+\varphi_{n2}\left(t\right).$$

**Figure 2:**
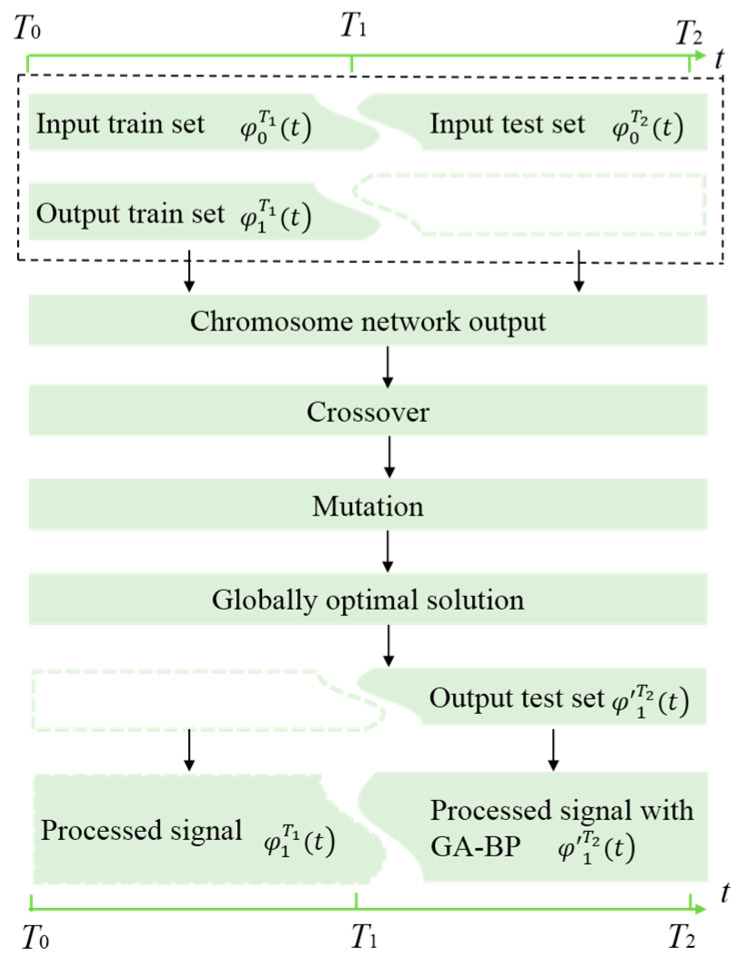
Algorithm flow chart for GA-BP.

The monitoring sensor phase $\varphi_{1}^{T_{1}}(t)$ and the reference sensor phase $\varphi_{0}^{T_{1}}(t)$ at time [*T*_0_, *T*_1_] is set as the train set, and the monitoring sensor phase $\varphi_{1}^{T_{2}}(t)$ and the reference sensor phase $\varphi_{0}^{T_{2}}(t)$ at time interval [*T*_1_, *T*_2_] is designated as the test set, and input into the GA-BP algorithm. The algorithm flow chart is shown in Figure 2.

The train set and the test set of the reference sensor serve as the input and the train set and the test set of the monitoring sensor serve as the output, where [*T*_0_, *T*_1_] is the original output for training and [*T*_1_, *T*_2_] is the predicted data. Firstly, the sample number of the train set is determined, the initial weight threshold is generated randomly, and the corresponding chromosome network output can be obtained through the calculation of the input samples by the network. Then, the fitness function is used to calculate the chromosome fitness and achieve regeneration, crossover, and variation. After the termination condition is reached, the global optimal solution can be obtained and the result can be output [30]. For the GA-BP-based noise suppression algorithm, there are two inputs and one output, so selection of the hidden layer can be determined by the two according to the empirical formula, and the hidden layer can be expressed as

(7) $$z_{k}=f_{1}\left(\omega_{1k}x_{1}-\beta_{k}\right),$$

where *k* is the number of hidden layer nodes, which can be estimated by the number of nodes in the input layer and the output layer, the number of input layer nodes is set according to the number of features in the input data, and the number of output layer nodes is set according to the number of predicted targets. *ω*_1*k*_ is the weight value from the input layer for the hidden layer. The weight from input layer to hidden layer is one of the core parts of neural network. These weights can be optimized using genetic algorithms. Specifically, the ownership values and thresholds of the network can be represented as a set of ordered chromosomes, expressed as real variables of the corresponding dimension according to the number of weights and thresholds. *x*_1_ is the training set phase; it has the monitoring sensor phase $\varphi_{1}^{T_{2}}(t)$ and the reference sensor phase $\varphi_{0}^{T_{2}}(t)$ at time [*T*_1_, *T*_2_]. *β_k_* is the offset from the input layer to the hidden layer; the offset of the hidden node can also be optimized by real coding. *f*_1_(·) is the transfer function of the hidden layer; the transfer function of the hidden layer generally adopts the Sigmoid function or the Tansig function. The output results layer is calculated by

(8) $$y_{j}=f_{2}\left(\sum_{k=1}^{N}\omega_{kj}b_{k}-\beta_{j}\right),$$

where *y_j_* is the result of the low-noise output hydroacoustic sensor obtained based on GA-BP, *j* the number of output layer nodes, and *b_k_* is the input vector value of the hidden layer. Following these steps, we can obtain the predicted monitoring sensor data $\varphi_{1}^{’T_{2}}(t)$ at the time interval [*T*_1_, *T*_2_].

## 3. Experimental Setup and Results

**Figure 3:**
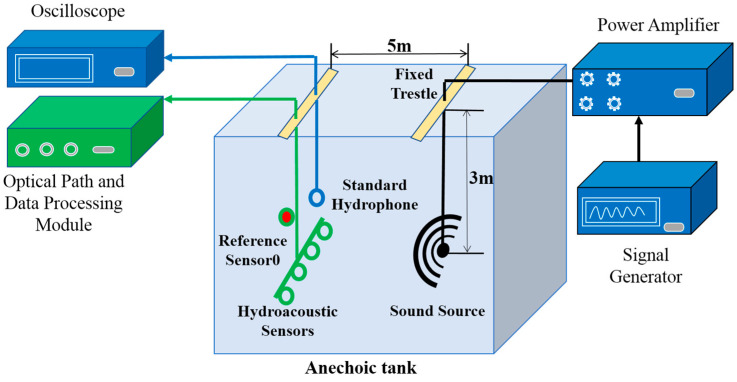
The experimental setup of GA-BP-based low-noise FBG hydroacoustic monitoring system.

The experimental setup of the GA-BP-based low-noise FBG hydroacoustic monitoring system is illustrated in Figure 3. The entire experiment is conducted in the anechoic tank with dimensions of 10 m in length, 10 m in width, and 5 m in depth, respectively. The signal generator outputs an arbitrary signal, which is then amplified by a power amplifier to drive the sound source and produce hydroacoustic signals. Both the hydroacoustic sensor and the reference sensor are placed at a depth of 3 m, with a 5 m spacing between the hydrophones and the sound source. A standard hydrophone is also deployed at a close location to calculate the effect of the proposed method on the sound pressure sensitivity of the fiber-optic hydrophone quantitatively. The standard hydrophone amplitude is read by an oscilloscope directly.

Additionally, the optical path is built according to the schematic diagram shown in Figure 1. The central wavelength of continuous light is 1550.1 nm, the modulation frequency of AOM is 100 kHz, the reflectance of the grating is −30 dB, the PD bandwidth is 200 MHz, and the sampling rate of the acquisition card is set to 250 MHz. The signals from the reference sensor and the monitoring sensor are obtained using the same phase demodulation algorithm. To simulate the sea environmental noise, the effective signal of the sound source is set as a pulse signal. Some Gaussian white noise signal is added to simulate ocean background noise. The spacing of adjacent sensors is set as 0.2 m. The sensor is wound into a ring with a radius of 6 cm to facilitate signal monitoring. The reference sensor is placed in a multi-layer vibration isolation closed box to minimize the interference from hydroacoustic signals.

**Figure 4:**
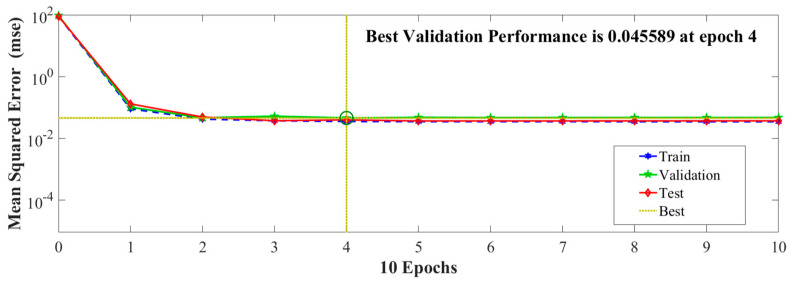
Training error of signal in the time domain of 0~6 ms.

According to the GA-BP algorithm flow chart, GA includes three main operating mechanisms: initial population, fitness function, selection, crossover, and mutation. The parameters of the genetic algorithm are binary coded and the initial population is generated randomly. The number of evolutions, population size, crossover probability, and mutation probability are set at 50, 5, 0.7, and 0.05, respectively. The time domain signals of 0~6 ms are used as train data, and the signal of 6~10 ms is used as test data. The mean square error (MSE) obtained from the train data, validation data, and test data is shown in Figure 4. The train set is used to learn the model and adjust the parameter weights continuously to achieve accurate predictive output results. The validation set is primarily used to adjust the model hyperparameters during training, preventing overfitting and determining the optimal model configuration. The test set is used for the final evaluation of the model to measure its generalization ability. As can be seen from Figure 4, the algorithm can achieve stable forward propagation and backpropagation in each epoch, and the three data sets converge well after multiple epochs. After 4 training epochs of the GA-BP algorithm, the training MSE error is stable at 0.045589. Considering the two factors of training period and error, the low-noise hydroacoustic signal reconstruction model is established with the time domain signal of 0~6 ms as the train data.

**Figure 5:**
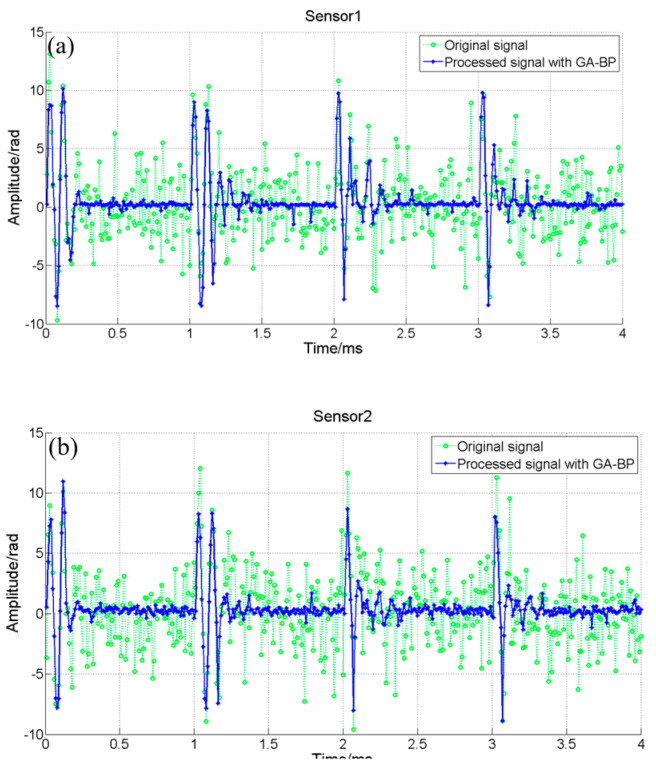

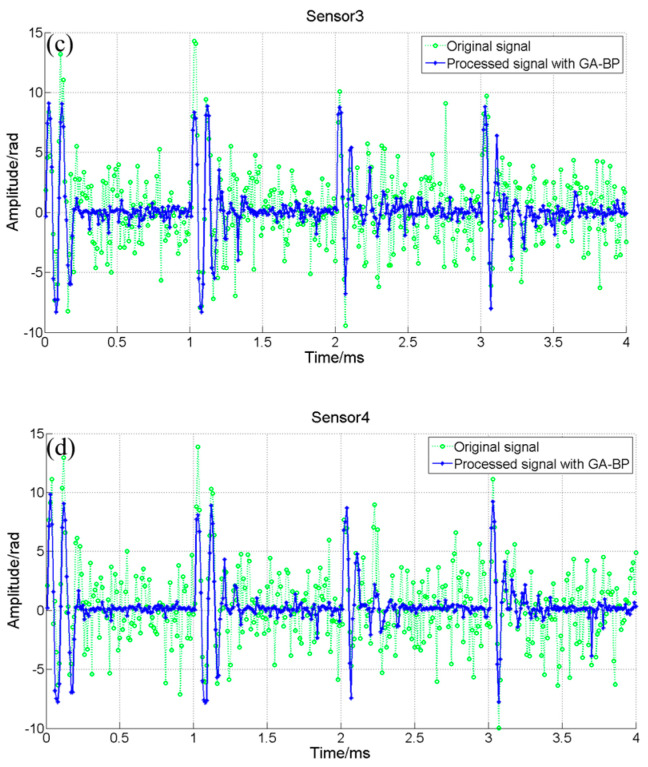
The time domain of the signal with 0.05 V noise for sensor 1~sensor 4. (**a**) sensor 1, (**b**) sensor 2, (**c**) sensor 3, (**d**) sensor 4.

The time domain diagram of reconstruction using our proposed algorithm is shown in Figure 5, where the solid blue line is the signal of the sensor reconstructed by the GA-BP algorithm within 6~10 ms, and the dotted green line is the original output signal of the hydroacoustic sensor without any processing within 6~10 ms. As can be seen from Figure 5, after learning 0~6 ms data by the GA-BP algorithm, the noise of 6~10 ms output results is reduced, and the signal-to-noise ratio is improved significantly. However, denoising methods usually introduce signal deformation along with SNR improvement; the correlation coefficient between the time domain signal and the piezoelectric hydrophone signal before and after GA-BP optimization is compared and analyzed. The correlation coefficient between the time domain signal and the piezoelectric hydrophone signal obtained by direct demodulation is 0.6872, whereas after the application of the GA-BP algorithm, the correlation coefficient reaches 0.9217, which demonstrates that the noise reduction algorithm has significant advantages in restoring effective signals in complex environments without introducing waveform distortion.

**Figure 6:**
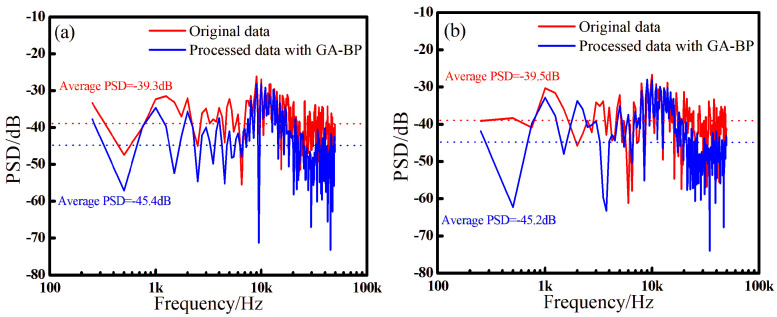

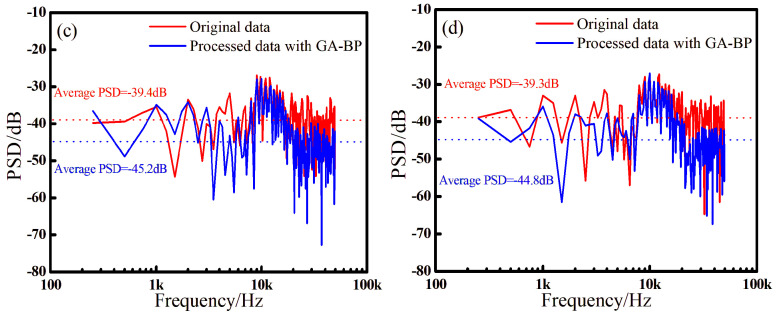
The frequency domain of the signal with 0.05 V noise for sensor 1~sensor 4. (**a**) sensor 1, (**b**) sensor 2, (**c**) sensor 3, (**d**) sensor 4.

The spectrum analysis of the signal in the 4 ms time window shown in Figure 5 is performed, and the results are displayed in Figure 6, where the results of the four sensors corresponded to (a), (b), (c), and (d), respectively. Since the duration of a pulse signal is very short in the time domain, it contains a wide range of frequency components from low to high frequency, making its spectrum typically broadband. To quantify the noise suppression of the proposed method, we use the average power spectral density (PSD), which describes the distribution of signal power over the frequency domain, to calculate the average power per unit frequency range. Due to the short duration of the pulse signal, the line spectrum results in the spectrum calculation are not accurate. Therefore, we perform statistics on the PSD across the full frequency band, and the average PSD calculated by the original signals of the four sensors is −39.3 dB, −39.5 dB, −39.4 dB, −39.3 dB, respectively. After applying the improved algorithm proposed in this paper, the average PSD is −45.4 dB, −45.2 dB, −45.2 dB, −44.8 dB, respectively, reducing the noise level by 6.1 dB, 5.7 dB, 5.8 dB, 5.1 dB, which proves that the demodulation consistency of the four sensors is relatively good and that the proposed algorithm has clear advantages in noise suppression. Additionally, it can be found that the proposed algorithm has better effect in high-frequency noise suppression, which may be due to the good signal isolation of the reference sensor in the high-frequency part; on the other hand, it may be related to the parameter selection of the algorithm itself.

Since we use a pulsed hydroacoustic signal in the experiment, there is a relationship between its spectral intensity and the pulse width, responder bandwidth and amplitude. In general, unlike single-frequency signals, when the pulse signal amplitude is steep, its spectrum amplitude declines slowly, and the effective frequency bandwidth becomes wider. Since the signal is not composed of a single frequency, for example, Sensor 1, Sensor 2, Sensor 3 and Sensor 4 in Figure 6 all exhibit deep valleys. Therefore, in some frequencies, amplitude anomaly is normal.

**Figure 7:**
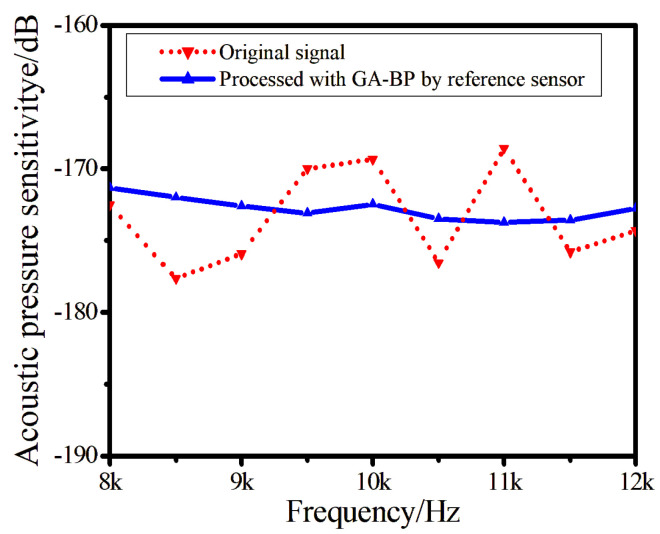
Acoustic pressure sensitivity results with different methods.

Figure 7 shows the sound pressure sensitivity calculated by the two methods within the bandwidth of 8 kHz to 12 kHz. The sound pressure sensitivity of the original signal ranges from −177.63 dB to −168.58 dB, with a response flatness of less than 9.05 dB. After processing based on the GA-BP algorithm using the reference sensor, the sound pressure sensitivity is within the range of −173.76 dB to −171.33 dB. When response flatness is less than 2.43 dB, the amplitude calculation error caused by noise interference is reduced after processing by our proposed algorithm, resulting in more stable response flatness, proving that the corrected output signal is more stable.

To verify the ability of the proposed algorithm to suppress signal noise in harsh environment, we generate noise of different amplitude through sound source simulation and calculate the power spectrum by using two methods and the average power spectrum amplitude within the bandwidth, respectively. As seen in Table 1, after processing with our GA-BP-based noise duration algorithm, the noise suppression effect under different amplitudes is quite significant. As the noise amplitude increases, the average PSD of the monitoring sensor decreases significantly, even though the noise itself continues to rise. After the algorithm processing, the noise amplitude is suppressed to a certain extent. In the case of bad SNR, the noise suppression effect is obvious, which can reach more than 18.3 dB.

**Figure 8:**
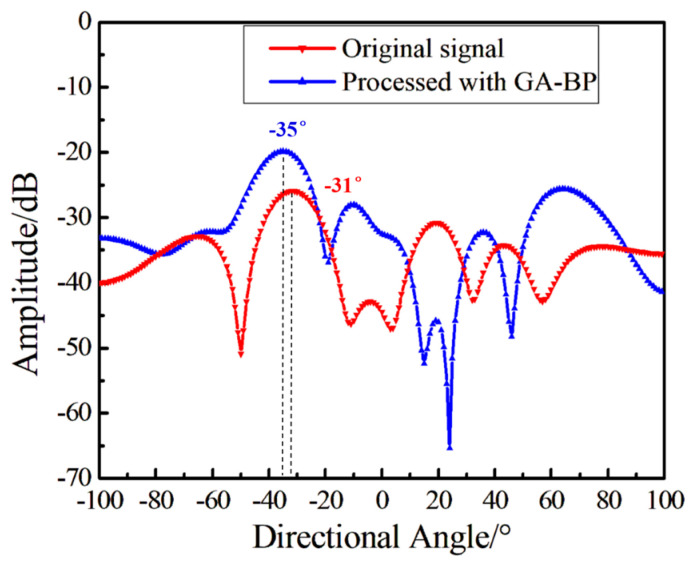
The directional result when the angle between the array and the sound source is −35°.

**Figure 9:**
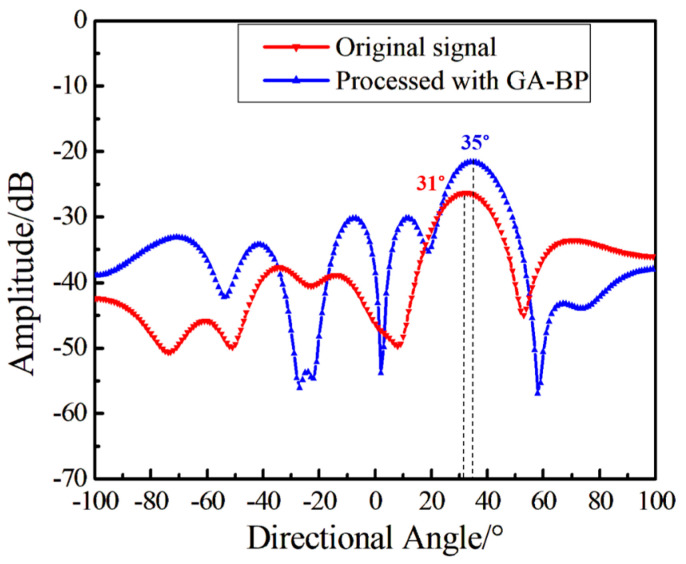
The directional result when the angle between the array and the sound source is 35°.

The above content provides a comparative analysis of the hydroacoustic indicators of a single sensor. To further verify the improvement of the proposed method on the synchronous processing of array signals, 11 sensors are selected to form an underwater monitoring array. The first sensor acts as a reference, while the remaining 10 sensors constitute the array. The hydrophones are arranged in a ring with a diameter of 6 cm and fixed on the cross bar, and the hydroacoustic transducer is used to send out short pulses for directional testing. During the test, the rotation angle of the transverse rod is, respectively, −35° and 35° with the sound source, and the results are shown in Figure 8 and Figure 9. From the time-domain results shown in Figure 5, it is evident that when the demodulation SNR is low, the accuracy and strength of the directional angle is affected by noise randomly. After the array is processed using a beamforming algorithm, the directivity accuracy and amplitude of the signal pair containing noise are affected. After GA-BP follow-up processing by the reference sensor, the directivity of the array is more accurate. The amplitude is more ideal, which demonstrates the advantages of the proposed method.

The above results demonstrate the significance of constructing input signals based on reference sensors from the time domain, frequency domain, and array signal processing of a single sensor. To further verify the generality of the signal generated by the reference structure in other GA-optimized neural network algorithms, we select the direct demodulation algorithm, the LSTM algorithm, and the GA-BP algorithm for comparative analysis. Five of the sensor arrays are selected for analysis, and the three algorithms are applied to the hydrophone array demodulation system to calculate the average PSD of hydroacoustic signals by three methods, the running time of array signal processing, and the directivity effect, respectively, representing the noise suppression effect, efficiency, and array beamforming ability. The results are shown in Table 2.

From the above results, it is evident that both LSTM and GA-BP algorithms effectively improve the signal-to-noise ratio compared to the original direct demodulation algorithm, proving the effectiveness of our proposed reference sensor optical path structure. Each algorithm has its own advantages and disadvantages. From the perspective of running time, the LSTM algorithm takes a long time in the training process due to its complex structure and the need for a large amount of data [27]. The GA-BP algorithm optimizes the BP neural network using a genetic algorithm, improves the prediction accuracy and convergence speed of the model, and the time is relatively short. The results show that the two improved neural network algorithms are superior to the original algorithm in directivity results, and the beamforming angle results are the same. Therefore, in summary, due to the characteristics of hydroacoustic signals in time sequence, the signals before and after are independent of each other. Considering the result and efficiency, the GA-BP algorithm is more optimal.

## 4. Conclusions

We propose a GA-BP-based low-noise FBG hydroacoustic monitoring system with a reference sensor. By constructing reference sensors, we obtain the train set and the test set of the GA-BP algorithm through data at different times and verify the rationality of the method through theoretical analysis and an acoustic pool experiment. The results show that the noise suppression effect is obvious under the condition of low SNR, which can reach more than 18.3 dB. The advantages of the proposed algorithm in array signal processing are also demonstrated by the directivity bluntness. Therefore, the results of low-noise output signals are greatly affected by the parameters of the algorithm model itself. In the subsequent experiments, we will continue to improve the algorithm design to further improve the noise suppression effect.

## Figures and Tables

**Table 1:** Average PSD in different noise amplitudes.

Noise Amplitude	0.01 V	0.05 V	0.1 V	0.2 V	0.5 V	1.0 V
Original Average PSD	−48.3 dB	−39.3 dB	−35.3 dB	−23.7 dB	−16.2 dB	−7.9 dB
Processed Average PSD	−49.2 dB	−45.4 dB	−41.2 dB	−33.6 dB	−30.5 dB	−26.2 dB
Gain	0.9 dB	6.1 dB	5.9 dB	9.9 dB	14.3 dB	18.3 dB

**Table 2:** Comparison table of sensors based on different algorithms.

Algorithms	Results	Sensor 1	Sensor 3	Sensor 5	Sensor 7	Sensor 9
Original signal	Average PSD	−39.3 dB	−39.1 dB	−38.7 dB	−37.2 dB	−37.9 dB
	Run Time	537 ms	536 ms	537 ms	537 ms	536 ms
	Directional Result	31°				
LSTM	Average PSD	−46.4 dB	−47.2 dB	−46.6 dB	−47.5 dB	−47.2 dB
	Run Time	5137 ms	5140 ms	5138 ms	5149 ms	5133 ms
	Directional Result	35°				
GA-BP	Average PSD	−45.4 dB	−46.1 dB	−45.1 dB	−44.9 dB	−45.7 dB
	Run Time	1583 ms	1535 ms	1554 ms	1530 ms	1596 ms
	Directional Result	35°				

## Data Availability

Data is unavailable due to privacy or ethical restrictions.

## References

[1] Tian W., Rui G., Liu G., Dong D. (2019). Efficient Acquisition Method for Marine Monitoring Data Based on Compressed Sensing. IEEE Access.

[2] Huang X., Pascal R.W., Chamberlain K., Banks C.J., Mowlem M., Morgan H. (2011). A Miniature, High Precision Conductivity and Temperature Sensor System for Ocean Monitoring. IEEE Sens. J..

[3] Yang Z., Li C., Chen F., Liu C., Cai Z., Cao W., Li Z. (2022). An in situ analyzer for long-term monitoring of nitrite in seawater with versatile liquid waveguide capillary cells: Development, optimization and application. Mar. Chem..

[4] Yuan D., Chen P., Mao Z., Zhang X., Zhang Z., Xie C., Zhong C., Qian Z. (2021). Ocean mixed layer depth estimation using airborne Brillouin scattering lidar: Simulation and model. Appl. Opt..

[5] Sutton J.N., Liu Y.W., Ries J.B., Guillermic M., Ponzevera E., Eagle R.A. (2018). δ^11^B as monitor of calcification site pH in divergent marine calcifying organisms. Biogeosciences Discuss..

[6] Zhang Q., Da L., Wang C., Yuan M., Zhang Y., Zhuo J. (2023). Passive ranging of a moving target in the direct-arrival zone in deep sea using a single vector hydrophone. J. Acoust. Soc. Am..

[7] Rong T., Wang C.X. (2023). Research on a stacked high-sensitivity hydroacoustic transducer. Sens. Rev..

[8] Wu S., Qiao Q., Liu G., Tan H., Zhang G., Zhang W., Wang R. (2023). MEMS co-vibration combined hydrophone. Measurement.

[9] Ramirez A., Quevedo L. (2022). Fiber-Optic Sensors Evaluate Well Performance in Polymerflooding Pilot. J. Pet. Technol..

[10] Wang J., Fu X., Gao H., Gui X., Wang H., Li Z. (2022). FPGA-Based Dynamic Wavelength Interrogation System for Thousands of Identical FBG Sensors. Photonics.

[11] Yan G., Pang Y., Gu H., Wu S., Li B., Liu W., Liu H., Liu C., Huang J. (2024). Detection of distorted interference pulses for UWFBG array based on odd function decomposition. Opt. Fiber Technol..

[12] Gui X., He S., Wang Y., Fu X., Guo Y., Li Z. (2023). Anti-noise UWFBG-array enhanced DAS system using double-pulse-based time-domain adaptive delay interference. Opt. Lett..

[13] Wang J., Li Z., Yang Q., Fu X., Gui X., Wang C., Wang H. (2019). Interrogation of a large-capacity densely spaced fiber Bragg grating array using chaos-based incoherent-optical frequency domain reflectometry. Opt. Lett..

[14] Wu S., Gu H., Pang Y., Liu W., Wang J., Huang J. (2023). Ultrathin interferometric hydrophone towed line array based on uwFBG. AIP Adv..

[15] Park J., Haralabus G., Zampolli M., Metz D. (2023). Low frequency ambient noise dynamics and trends in the Indian Ocean, Cape Leeuwin, Australia. J. Acoust. Soc. Am..

[16] Wang L., Wang Q. (2016). The influence of marine biological noise on sonar detection. Proceedings of the 2016 IEEE/OES China Ocean Acoustics (COA).

[17] Radford C.A., Jeffs A.G., Tindle C.T., Montgomery J.C. (2008). Temporal patterns in ambient noise of biological origin from a shallow water temperate reef. Oecologia.

[18] Meng K., Liu P., Chen H., Ren H.G. (2007). A fiber optic hydrophone with chaos. J. Harbin Eng. Univ..

[19] Ma F., Chen K., Zhao Y., Zhu F., Guo M., Tian Y., Yuan X., Ma Y., Hang C. (2022). Fiber-Optic Photoacoustic Sensing Probe Capable of Resisting Interference form Ambient Noise, and Sensing System.

[20] Cai Y., Yu Z., Mo D., Liu R., Chen A., Dai B., Li Y. (2020). Noise reduction with adaptive filtering scheme on interferometric fiber optic hydrophone. Optik.

[21] Pang Y., Liu H., Zhou C., Huang J., Gu H., Zhang Z. (2022). Pretreatment of Ultra-Weak Fiber Bragg Grating Hydrophone Array Based on Cubic Spline Interpolation Using Intensity Compensation. Sensors.

[22] Moradi H., Hosseinibalam F., Hassanzadeh S. (2019). Simulation and experimental investigation about interferometric optical fiber acoustic sensor for sensitivity enhancement. Measurement.

[23] Peng Y., Xiang W. (2020). Short-term traffic volume prediction using GA-BP based on wavelet denoising and phase space reconstruction. Phys. A Stat. Mech. Its Appl..

[24] Wu Y., Yang R., Guo C., Yang R. (2019). GA-BP Neural Network Based Intensity Compensation for Optical Fiber Displacement Sensor. Electron. Opt. Control.

[25] Liu H., Zhou C., Pang Y., Fan D., Chen X. High Spatial Resolution Reconstruction of Hydroacoustic Signal from Drawing Tower Grating with Long Cavity Based on GA-BP. Proceedings of the 2021 International Conference of Optical Imaging and Measurement (ICOIM).

[26] Ma Y., Zhang H., Dai X., Zhang J., Gong Y., Helen L., Ye B. (2024). High precision three-dimensional ellipse fitting correction for galloping monitoring. AIP Adv..

[27] Liu H., Zhou C., Pang Y., Chen X., Pan Z., Wang L., Fan D. (2023). Temperature Demodulation for an Interferometric Fiber-Optic Sensor Based on Artificial Bee Colony–Long Short-Term Memory. Photonics.

[28] Anand V.R., Ramanan S.R., Santhanakrishnan T., Asokan S. (2023). Side Hole Packaged Shell-Encapsulated Etched FBG Hydrophone. IEEE Sens. J..

[29] Pu H., Song X., Tian Y., Wang M., Sun Y., Peng Y., Luo J., Ding J. (2024). Sensor Fusion for Active Vibration Isolation System with Double Noise: An Adaptive Kalman Filter Approach. IEEE Trans. Ind. Electron..

[30] Zhu C., Zhang J., Liu Y., Ma D., Li M., Xiang B. (2020). Comparison of GA-BP and PSO-BP neural network models with initial BP model for rainfall-induced landslides risk assessment in regional scale: A case study in Sichuan, China. Nat. Hazards.

